# Supplementary material for: Assessing the effectiveness of a national protected area network for carnivore conservation
Source: Nat Commun. 2020 Jun 11;11:2957. doi: 10.1038/s41467-020-16792-7 (PMC7289803; doi:10.1038/s41467-020-16792-7)
Supplement: Supplementary file 1 — Supplementary Information [file 41467_2020_16792_MOESM1_ESM.pdf]

Assessing the effectiveness of a national protected area network for carnivore conservation

Terraube et al.

Supplementary Tables

Supplementary Table 1: Number and size of PAs in each Game Management Area (4 clusters), included in the analyses of PA effectiveness.

**Game Management Area**	**Number of PAs**	**Average size of PAs (km^2) ± SD**
South	80	8.76 ± 42.86
Central	145	58.16 ± 416.57
North	113	25.33 ± 59.47
Lapland	65	538.79 ± 837.81

Supplementary Table 2: Number of paired units per Game Management Area (4 clusters) included in matching analyses.

**Years**	**Number of paired units (1 inside PA-1 outside PA) per Game Management Area (4 clusters)**				**Total Number of paired units**
	South	Central	North	Lapland	
1989	8	17	5	6	36
1990	31	48	33	23	135
1991	39	70	36	30	175
1992	37	56	39	35	167
1993	41	67	39	38	185
1994	34	59	36	36	165
1995	35	57	37	36	165
1996	37	42	34	36	149
1997	29	37	37	30	133
1998	26	35	30	29	120
1999	34	39	29	21	123
2000	30	47	21	23	121
2001	25	41	21	19	106
2002	21	38	22	21	102
2003	23	37	21	20	101
2004	26	39	21	24	110
2005	26	43	22	20	111
2006	26	44	17	16	103
2007	24	29	18	11	82
2008	18	28	16	14	76
2009	27	40	19	17	103
2010	10	24	12	9	55
2011	11	28	14	7	60
2012	9	30	12	12	63
2013	19	34	15	15	83
2014	5	20	8	11	44
2015	12	31	18	18	79
2016	19	33	15	17	84
2017	12	34	14	15	75
	**694**	**1147**	**661**	**609**	**3111**

Supplementary Table 3: Estimates of the absolute PA effect (all regions and all years pooled) for four species of large carnivores in Finland over the study period: 1989-2017. Median values and 95% CI obtained through iterative random sampling (see Methods) are presented below. The number of imputations corresponds to the product of the number of all observations per pair of matched units.

**Species**	**Absolute PA Effect (Median)**	**95% CI**	**Number of imputations**
Brown bear	-0.310996	-0.516, -0.0831	9.001727e+60
Eurasian lynx	-0.07742175	-0.255, 0.085	3.918726e+74
Gray wolf	0.01789073	-0.031, 0.077	70778880
Wolverine	0.09759216	-0.044, 0.225	3.06491e+16

Supplementary Table 4: Mean annual estimates of the absolute PA effect (network level) for four species of large carnivores in Finland over the study period: 1989-2017.

Species	Year	Absolute PA Effect (Median)	95% CI	p-value	Bonferroni-adjusted p-values
**Brown bear**	1988	1.410	1.418, 1.427	0.04	1
	1989	-0.018	-4.259, 2.420	0.441	1
	1990	1.388	-12.814, 13.611	0.907	1
	1991	-1.384	-6.240, 4.252	0.268	1
	1992	-1.393	-11.843, 8.587	0.632	1
	1993	-1.325	-7.024, 4.157	0.189	1
	1994	-1.415	-2.927, 3.562	0.244	1
	1995	1.400	-8.465, 4.801	0.916	1
	1996	-1.387	-6.399, 9.515	0.555	1
	1997	-1.352	-10.066, 5.364	0.585	1
	1998	1.380	-5.627, 12.601	0.696	1
	1999	-1.402	-7.085, 3.518	0.229	1
	2000	-1.443	-7.331, 7.386	0.570	1
	2001	-1.365	-7.360, 5.352	0.263	1
	2002	1.421	-5.873, 4.170	0.956	1
	2003	-1.299	-2.653, 2.810	0.933	1
	2004	-1.410	-6.906, 2.824	0.214	1
	2005	1.453	-3.807, 3.798	0.320	1
	2006	1.377	-5.335, 1.516	0.690	1
	2007	1.383	-2.315, 4.180	0.319	1
	2008	-1.392	-7.821, 2.939	0.243	1
	2009	1.402	-3.079, 3.691	0.430	1
	2010	-1.391	-2.816, 1.566	0.076	1
	2011	-1.350	-7.062, 3.640	0.305	1
	2012	-1.410	-7.027, 3.616	0.156	1
	2013	1.366	-4.600, 5.438	0.721	1
	2014	-0.001	-4.377, 4.383	0.994	1
	2015	-1.379	-6.936, 4.188	0.252	1
	2016	-1.385	-9.620, 8.138	0.230	1
	2017	-1.380	-9.590, 9.907	0.473	1
**Eurasian lynx**					
	1989	-0.827	-3.992, 1.694	0.468	1
	1990	0.215	-1.970, 2.641	0.561	1
	1991	-0.834	-5.15, 1.519	0.173	1
	1992	-0.429	-1.469, 2.396	0.841	1
	1993	0.204	-1.674, 4.562	0.452	1
	1994	-0.422	-5.383, 0.902	0.055	1

	1995	-0.357	-3.973, 1.041	0.266	1
	1996	-1.107	-3.121, 1.550	0.041	1
	1997	-0.476	-2.301, 2.018	0.647	1
	1998	-0.834	-3.461, 1.285	0.049	1
	1999	-0.701	-2.950, 2.721	0.508	1
	2000	0.422	-8.285, 3.102	0.549	1
	2001	-0.416	-4.420, 3.229	0.684	1
	2002	-0.651	-3.704, 3.117	0.736	1
	2003	0.847	-3.761, 6.678	0.212	1
	2004	-0.412	2.615, 3.343	0.905	1
	2005	0.428	-2.180, 6.219	0.290	1
	2006	-0.449	-3.231, 2.524	0.244	1
	2007	-0.408	-2.325, 3.869	0.625	1
	2008	0.410	-2.511, 6.698	0.212	1
	2009	0.251	-3.891, 6.707	0.347	1
	2010	-0.157	-5.106, 3.355	0.299	1
	2011	0.716	-2.962, 4.247	0.054	1
	2012	0.212	-4.089, 4.359	0.907	1
	2013	0.281	-4.777, 3.426	0.793	1
	2014	0.819	-1.378, 4.707	0.076	1
	2015	0.417	-3.265, 2.485	0.947	1
	2016	0.116	-2.583, 1.898	0.962	1
	2017	0.0007	-2.094, 3.626	0.455	1
**Gray wolf**					
	1989	NA	NA	NA	NA
	1990	-0.834	-0.834, -0.834	NA	1
	1991	-2.096	-3.288, -0.905	0.343	1
	1992	6.309	1.116, 11.501	0.454	1
	1993	0.849	0.849, 0.849	NA	1
	1994	-0.470	-1.608, 0.688	0.762	1
	1995	0.642	-0.566, 1.611	0.318	1
	1996	-0.848	-0.905, 2.337	0.842	1
	1997	-0.837	-0.869, 2.254	0.990	1
	1998	-7.456	-7.456, -7.456	NA	1
	1999	1.245	0.855, 3.228	0.145	1
	2000	-0.695	-3.191, 1.731	0.519	1
	2001	-0.420	-0.420, -0.420	NA	1
	2002	-1.657	-8.830, 0.331	0.133	1
	2003	1.921	-0.735, 12,187	0.290	1
	2004	0.832	-0.330, 1.587	0.118	1
	2005	0.830	-1.497, 15.547	0.252	1
	2006	0.417	-0.775, 0.456	0.767	1
	2007	-0.448	-0.879, 0.730	0.385	1
	2008	0.424	0.424, 0.424	NA	1
	2009	-0.120	-1.565, 2.192	0.876	1
	2010	0.827	0.827, 0.827	NA	1

	2011	NA	NA	NA	NA
	2012	-0.312	-0.408, -0.217	0.198	1
	2013	-0.426	-0.433, -0.418	0.012	0.252
	2014	-0.415	-0.804, -0.159	0.052	1
	2015	-0.411	-7.386, 2.399	0.570	1
	2016	-0.840	-1.713, 0.698	0.234	1
	2017	0.621	0.428, 0.814	0.201	1
**Wolverine**					
	1989	NA	NA	NA	NA
	1990	NA	NA	NA	NA
	1991	NA	NA	NA	NA
	1992	-0.849	-0.860, -0.834	0.010	0.25
	1993	0.835	-0.775, 4.035	0.448	1
	1994	-0.849	-0.859, -0.838	0.008	0.20
	1995	0.433	-1.542, 1.982	0.455	1
	1996	0.333	-0.786, 0.798	0.696	1
	1997	-0.832	-1.567, 2.431	0.829	1
	1998	-1.683	-1.695, -0.798	0.038	0.95
	1999	1.281	-0.638, 1.718	0.100	1
	2000	-0.838	-2.372, -0.828	0.057	1
	2001	0.833	-0.756, 0.833	0.670	1
	2002	-0.418	-0.418, -0.418	NA	1
	2003	0.710	-1.677, 2.892	0.333	1
	2004	-0.703	-1.694, 1.466	0.299	1
	2005	0.834	-0.856, 2.350	0.210	1
	2006	0.428	-1.422, 0.826	0.647	1
	2007	-0.626	-1.593, 0.738	0.390	1
	2008	0.410	-1.064, 1.663	0.585	1
	2009	-0.635	-3.366, 0.804	0.125	1
	2010	-0.010	-1.591, 1.530	0.884	1
	2011	-0.418	-1.641, 0.393	0.217	1
	2012	-0.415	-1.076, 0.422	0.336	1
	2013	0.418	-1.788, 1.545	0.786	1
	2014	0.136	-1.195, 0.839	0.949	1
	2015	0.620	-2.193, 2.527	0.247	1
	2016	-0.316	-3.117, 0.830	0.325	1
	2017	-0.426	-1.961, 1.526	0.615	1

Supplementary Table 5: Mean annual estimates of the absolute PA effect (network level), per region (four clusters of the 15 Game Management Areas divided in South, Central, North Finland and Lapland) for four species of large carnivores in Finland over the study period: 1989-2017. Medians estimated without randomization due to small numbers of observation per pair of matched wildlife units are highlighted in bold. The number of imputations corresponds to the product of the number of all observations per pair of matched units.

**Region**	**Species**	**Absolute PA Effect (Median)**	**95% CI**	**Number of imputations**
**South**	Brown bear	**1.443698**	**-2.62, 2.731**	2
	Eurasian lynx	-0.3867036	-1.137, 0.432	2304
	**Gray wolf**	**-0.4482912**	**-1.671, -0.435**	2
	Wolverine	0.00	0.00	-
**Central**	Brown bear	-0.6339295	-1.363, 0.998	69120
	Eurasian lynx	-0.316251	-0.859, 0.429	1108800
	**Gray wolf**	**11.77399**	**-0.266, 18.258**	1
	Wolverine	**0.4191649**	**-2.087, 2.279**	4
**North**	Brown bear	-0.3420483	-1.037, 0.330	2400
	Eurasian lynx	0.3541406	-0.109, 1.336	520
	**Gray wolf**	**0.4166735**	**-1.721, 11.338**	8
	Wolverine	**0.2580538**	**-0.892, 1.089**	108
**Lapland**	Brown bear	-0.1491564	-0.707, 0.165	720
	Eurasian lynx	**-1.447016**	**-3.771, 0.463488**	8
	Gray wolf	-	-	-
	Wolverine	**0.5544381**	**-2.249, 1.580**	3

Supplementary Table 6: Model selection results (AIC, BIC and log.Lik values) for the factors explaining variations in densities of four carnivore species in Finland (including protection status). Model composition is described in Supplementary Methods.

	**AIC**	**BIC**	**log.Lik**
**BEAR**			
model 1	10913.36	11000.18	-5439.68
model 2	10912.54	11004.46	-5438.27
model 3	10914.56	11006.48	-5439.28
model 4	10913.25	11010.28	-5437.63
model 5	10845.66	**10942.68**	-5403.83
model 6	**10844.81**	10946.94	-5402.40
model 7	10846.88	10949.01	-5403.44
model 8	10846.80	10948.93	-5403.40
model 9	10845.54	10952.78	-5401.77
model 10	10845.66	10952.89	-5401.83
model 11	10847.85	10955.09	-5402.93
model 12	10846.10	10958.45	-5401.05
**LYNX**			
model 1	16543.43	16628.88	-8254.72
model 2	16543.61	16634.08	-8253.80
model 3	16539.62	16630.10	-8251.81
model 4	16541.28	16636.79	-8251.64
model 5	15640.43	15735.93	-7801.22
model 6	15640.64	15741.17	-7800.32
model 7	**15636.55**	**15737.08**	-7798.28
model 8	15642.35	15742.88	-7801.18
model 9	15638.23	15743.79	-7798.12
model 10	15642.52	15748.07	-7800.26
model 11	15638.54	15744.09	-7798.27
model 12	15640.20	15750.79	-7798.10
**WOLF**			
model 1	3613.69	3699.14	-1789.85
model 2	3613.98	3704.45	-1788.99
model 3	3615.49	3705.96	-1789.74
model 4	3616.88	3712.39	-1789.44
model 5	**3540.16**	**3635.66**	-1751.08
model 6	3542.34	3642.87	-1751.17
model 7	3540.58	3641.10	-1750.29
model 8	3540.79	3641.32	-1750.40
model 9	3542.76	3648.31	-1750.38
model 10	3544.72	3655.30	-1750.36
model 11	3542.41	3647.96	-1750.20
model 12	3544.31	3654.89	-1750.16
**WOLVERINE**			
model 1	4597.24	4682.69	-2281.62
model 2	4599.18	4689.65	-2281.59

model 3	4598.32	4688.80	-2281.16
model 4	4600.16	4695.66	-2281.08
model 5	4338.38	**4433.89**	-2150.19
model 6	4340.31	4440.84	-2150.16
model 7	4339.87	4440.40	-2149.93
model 8	**4336.79**	4437.32	-2148.40
model 9	4341.53	4447.09	-2149.77
model 10	4338.86	4444.42	-2148.43
model 11	4338.48	4444.03	-2148.24
model 12	4340.47	4451.05	-2148.24

Supplementary Table 7: Parameters of the best model selected describing the covariates (confounding covariates + PA + interactive effects between PA and confounding covariates) influencing the densities of each species of large carnivore in Finland, over the study period: 1989-2017 (hurdle mixed-effects model; Rizopoulos, 2019).

**Species**	**Covariates**	**Estimate**	**Std. Error**	**z-value**	**p-value**
**Brown bear**	Intercept	0.0653	0.0029	22.1993	<0.0001
	Terrain ruggedness	-0.0083	0.0022	-3.8072	0.00014053
	% Forest Cover	0.0164	0.0024	6.7793	<0.0001
	Dist. Settlement	0.0101	0.0035	2.8724	0.00407397
	Hum. Pop. Density	-0.0047	0.0010	-4.6264	<0.0001
	Latitude	-0.0243	0.0034	-7.1423	<0.0001
	Longitude	0.0738	0.0033	22.0537	<0.0001
	**PA**	**0.0065**	**0.0034**	**1.8754**	**0.06073735**
	Years	0.0129	0.0017	7.6160	<0.0001
	PA × Latitude	0.0034	0.0030	1.1614	0.24547315
**Eurasian lynx**	Intercept	0.0157	0.0048	3.2693	0.00107824
	Terrain ruggedness	0.0128	0.0034	3.7367	0.00018649
	% Forest Cover	-0.0061	0.0029	-2.1427	0.03213933
	Dist. Settlement	-0.0055	0.0025	-2.2160	0.02669057
	Hum. Pop. Density	-0.0018	0.0029	-0.6224	0.53366691
	Latitude	-0.0180	0.0041	-4.3505	<0.0001
	Longitude	-0.0016	0.0053	-0.2987	0.76520506
	**PA**	**0.0041**	**0.0066**	**0.6236**	**0.53291014**
	Years	0.0074	0.0032	2.3231	0.02017435
	**PA × Longitude**	0.0176	0.0069	2.5675	0.01024244
**Gray wolf**	Intercept	0.0011	0.0016	0.6798	0.496660
	Terrain ruggedness	-0.0012	0.0013	-0.9285	0.353158
	% Forest Cover	0.0003	0.0010	0.2904	0.771541
	Dist. Settlement	0.0034	0.0025	1.3498	0.177077
	Hum. Pop. Density	-0.0003	0.0006	-0.4985	0.618166
	Latitude	-0.0034	0.0019	-1.7707	0.076610
	Longitude	0.0018	0.0018	1.0456	0.295730
	**PA**	**0.0008**	**0.0024**	**0.3296**	**0.741677**
	Years	-0.0029	0.0015	-2.0026	0.045225
**Wolverine**					
	Intercept	-0.0015	0.0016	-0.9138	0.360826
	Terrain ruggedness	-0.0029	0.0014	-2.0982	0.035889
	% Forest Cover	0.0012	0.0012	0.9720	0.331044
	Dist. Settlement	-0.0025	0.0029	-0.8583	0.390740
	Hum. Pop. Density	0.0000	0.0003	-0.0222	0.982318

	Latitude	0.0038	0.0020	1.8521	0.064017
	Longitude	-0.0022	0.0013	-1.7091	0.087430
	**PA**	**-0.0018**	**0.0027**	**-0.6832**	**0.494464**
	Years	-0.0009	0.0019	-0.4632	0.643189
	**PA × Years**	**-0.0055**	**0.0027**	**-2.0294**	**0.042422**

Supplementary Table 8: Total number of units used in the two-part mixed-effects models versus total available units per Game Management Area (4 clusters).

**Years**	**Number of wildlife units per region used in the two part mixed-effect models/Total number of available units per region**				
	**South**	**Central**	**North**	**Lapland**	**Total**
1989	51/99	36/125	90/141	29/39	206/404
1990	87/184	114/245	178/283	113/152	492/864
1991	100/209	141/327	173/282	137/186	551/1004
1992	99/193	138/304	175/273	165/218	577/988
1993	104/208	163/343	174/271	170/223	611/1045
1994	97/185	155/336	155/247	151/201	558/969
1995	93/180	155/326	159/244	153/201	560/951
1996	91/182	150/316	116/193	143/196	500/887
1997	71/154	127/264	148/235	148/198	494/887
1998	78/153	130/264	142/220	134/183	484/820
1999	90/174	139/281	111/187	131/178	471/820
2000	91/178	131/281	108/183	121/162	451/804
2001	77/152	126/254	104/172	119/154	426/732
2002	73/149	130/267	100/176	106/149	409/741
2003	76/146	114/227	106/182	106/151	402/706
2004	93/164	130/260	110/188	106/148	439/760
2005	77/152	136/277	116/194	108/147	437/770
2006	83/381	127/295	95/177	102/141	407/994
2007	67/305	115/241	78/136	86/115	346/797
2008	55/263	122/265	85/152	101/133	363/813
2009	80/364	139/313	88/155	100/129	407/961
2010	60/251	91/209	69/112	85/103	305/675
2011	64/260	115/248	76/126	82/103	337/737
2012	59/235	107/226	69/119	92/121	327/701
2013	66/310	117/261	86/145	96/124	365/840
2014	35/108	96/199	69/107	90/116	290/530
2015	59/192	122/252	94/159	97/130	372/733
2016	75/256	131/287	98/168	90/122	394/833
2017	63/233	141/305	115/184	90/122	409/844

Supplementary Table 9: Summary of matching covariates used in quasi-experimental evaluation of the impacts of PAs on densities of 4 species of large carnivores in Finland.

**Matching covariates**	**Centroid/Buffer**	**Definition**	**Hypothesis**	**Source**
% Forest Cover	Buffer	% Forest cover within a 1km radius from the center of each wildlife unit	Large carnivores are expected to reach higher densities in units with a higher proportion of forest cover	Corinne Land Cover 2012.<br><br>https://www.syke.fi/en-US/Open_information/Spatial_datasets
Terrain ruggedness (sdElev)	Buffer	standard deviation of elevation (25m resolution)	Large carnivores are expected to reach higher densities in units located in more rugged landscapes (higher slope)	Alti.tif<br><br>https://www.maanmittauslaitos.fi/en/maps-and-spatial-data
Hum. Pop. Density	Centroid (bilinear interpolation from neighbouring grid points)	Number of people per square kilometer within a 1km radius from the center of each wildlife unit	Large carnivores are expected to reach higher densities in units where human population density is lower.	VAKI2016
Distance to the closest settlement<br><br>(Dist. Settl.)	Centroid	Distance between the center of each wildlife unit and the closest settlement	Large carnivores are expected to reach higher densities in units located further away from human settlements.	YKR1<br><br>https://www.syke.fi/en-US/Open_information/Spatial_datasets
Latitude		Latitude (Finnish coordinate system)	Latitude is associated to a productivity gradient in boreal ecosystems. We expect decreasing large carnivore densities along a south-north gradient in Finland.	
Longitude		Longitude (Finnish coordinate system)	Large carnivore are expected to reach higher at higher longitudes (eastern Finland) as the proximity of the Russian frontier guarantees increased influx of individuals from source Russian populations.	

Supplementary Figures

Supplementary Figure 1a: Assessment of matching effectiveness for the covariates Forest Cover, Latitude and Longitude.

Matching formula/MatchIt package:

matchit(formula = PA ~ ForCov + Longitude + Latitude + sdElev + PopDens + DistSettl, data = DataTriangPANAbis, method = "nearest", distance = "mahalanobis", replace = "TRUE", caliper = 0.2).

Q-Q plots of quantile differences in the distribution of matching covariates prior to matching and after matching. 1:1 line indicates perfect agreement between covariate distributions for wildlife units within vs outside of a protected area.

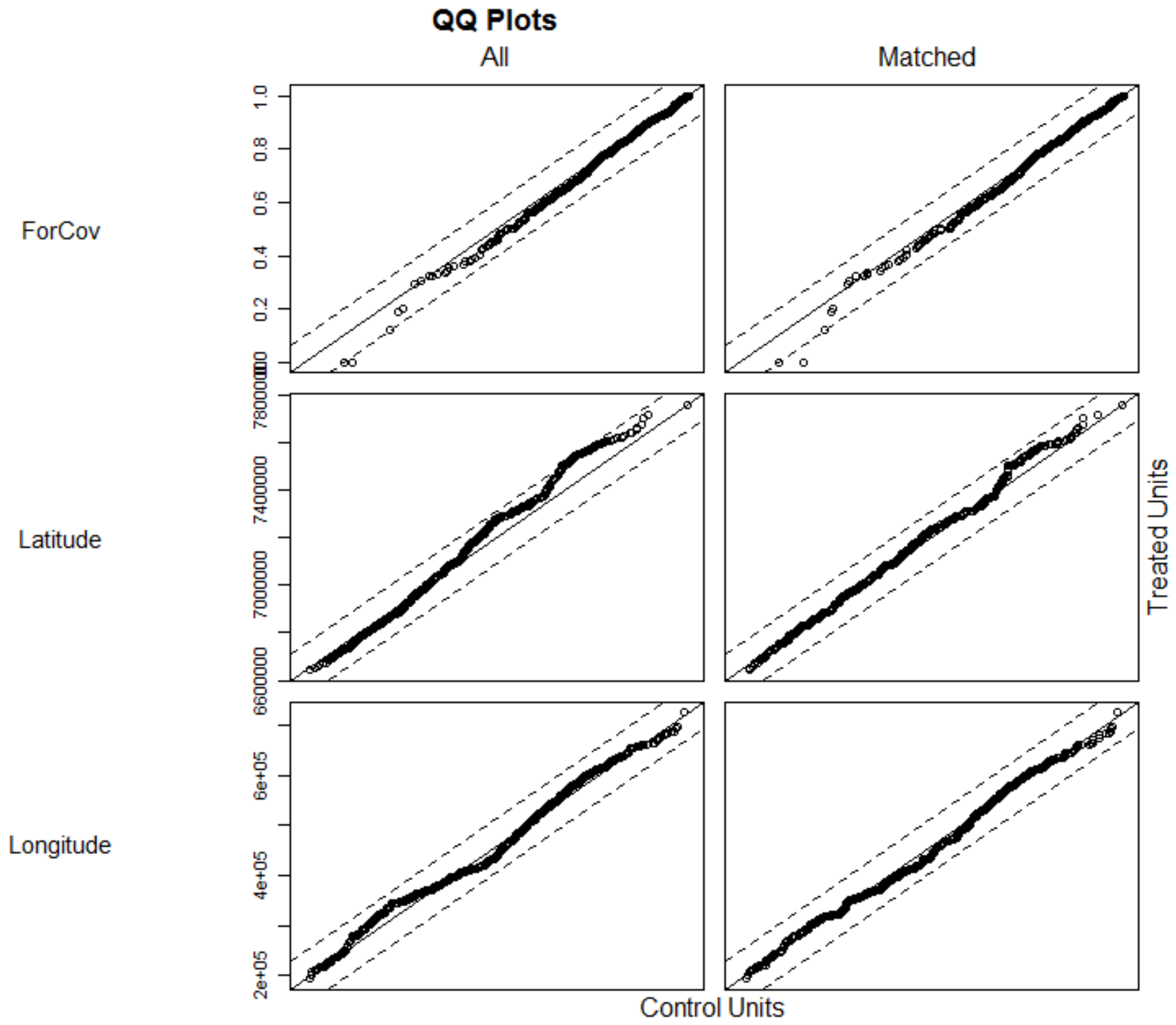
QQ Plots
All
Matched
ForCov
Latitude
Longitude
0.0
0.2
0.4
0.6
0.8
1.0
6600000
7000000
7400000
7800000
2e+05
4e+05
6e+05
Control Units
Treated Units

Supplementary Figure 1b. Assessment of matching effectiveness for the covariates 'slope', 'Human population density' and 'Distance to the closest settlement'. Q-Q plots of quantile differences in the distribution of matching covariates prior to matching and after matching. 1:1 line indicates perfect agreement between covariate distributions for wildlife units within vs outside of a protected area.

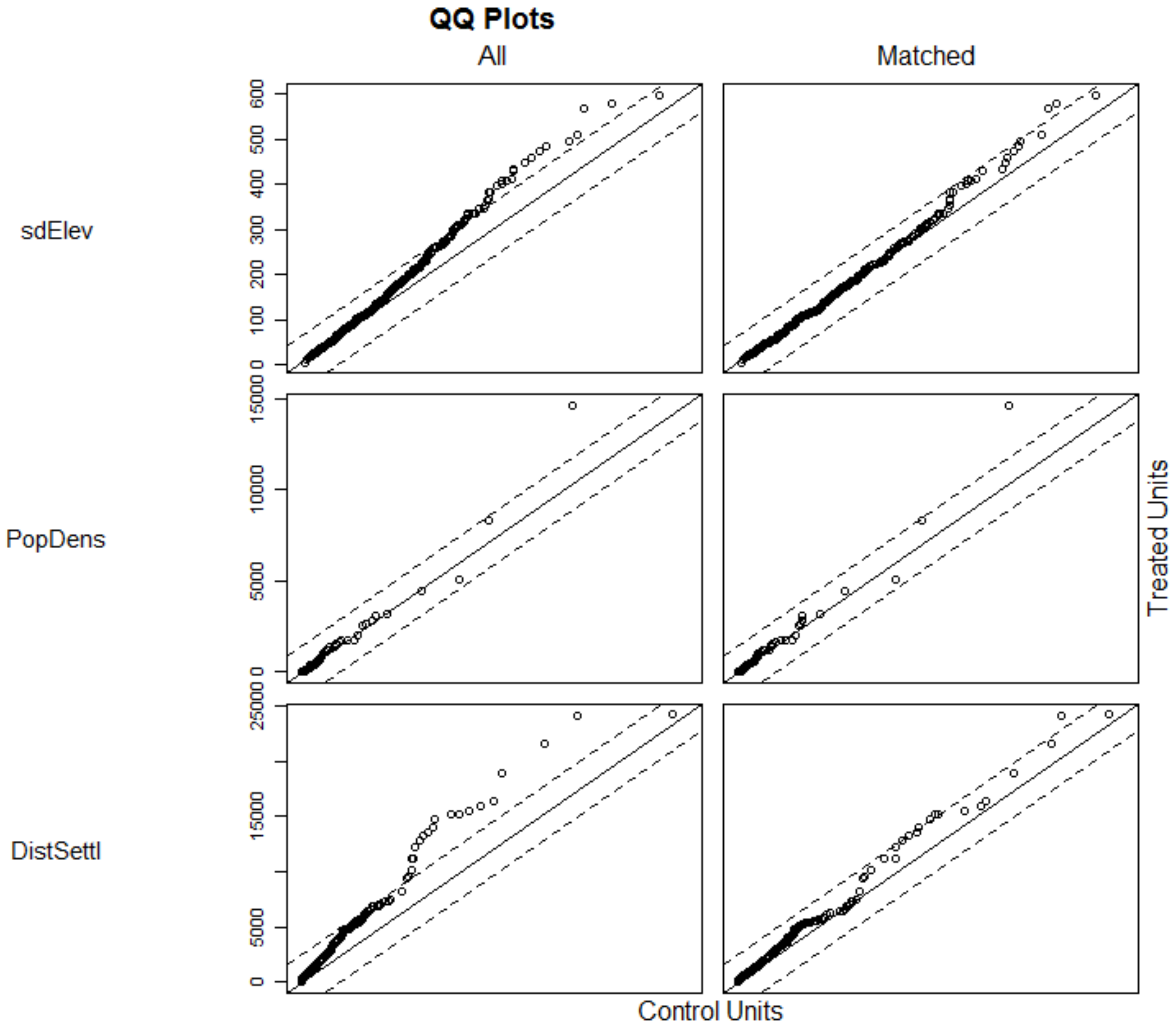

Supplementary Methods:

Structure of the 12 candidate models that were evaluated for each large carnivore species (two-part mixed effects models, GLMMadaptive package).

model1: Density ~ sdElevs + ForCovs + DistSettls + PopDenss + LatitudeNums + LongitudeNums + PA.f , random = ~1 |TriangleID.f.

model2: Density ~ sdElevs + ForCovs + DistSettls + PopDenss + LatitudeNums + LongitudeNums + PA.f + PA.f:LatitudeNums , random = ~1 |TriangleID.f.

model3: Density ~ sdElevs + ForCovs + DistSettls + PopDenss + LatitudeNums + LongitudeNums + PA.f + PA.f:LongitudeNums , random = ~1 |TriangleID.f.

model4: Density ~ sdElevs + ForCovs + DistSettls + PopDenss + LatitudeNums + LongitudeNums + PA.f + PA.f:LatitudeNums + PA.f:LongitudeNums , random = ~1 |TriangleID.f.

model5: DensBear ~ sdElevs + ForCovs + DistSettls + PopDenss + LatitudeNums + LongitudeNums + PA.f + years, random = ~1 |TriangleID.f,

model6: Density ~ sdElevs + ForCovs + DistSettls + PopDenss + LatitudeNums + LongitudeNums + PA.f + years + PA.f:LatitudeNums, random = ~1 |TriangleID.f.

model7: Density ~ sdElevs + ForCovs + DistSettls + PopDenss + LatitudeNums + LongitudeNums + PA.f + years + PA.f:LongitudeNums, random = ~1 |TriangleID.f.

model8: Density ~ sdElevs + ForCovs + DistSettls + PopDenss + LatitudeNums + LongitudeNums + PA.f + years + PA.f:years, random = ~1 |TriangleID.f.

model9<- Density ~ sdElevs + ForCovs + DistSettls + PopDenss + LatitudeNums + LongitudeNums + PA.f + years + PA.f:LatitudeNums + PA.f:LongitudeNums , random = ~1 |TriangleID.f.

model10: Density ~ sdElevs + ForCovs + DistSettls + PopDenss + LatitudeNums + LongitudeNums + PA.f + years + PA.f:LatitudeNums + PA.f:years, random = ~1 |TriangleID.f.

model11: Density ~ sdElevs + ForCovs + DistSettls + PopDenss + LatitudeNums + LongitudeNums + PA.f + years + PA.f:LongitudeNums + PA.f:years, random = ~1 |TriangleID.f.

model12: Density ~ sdElevs + ForCovs + DistSettls + PopDenss + LatitudeNums + LongitudeNums + PA.f + years + PA.f:LatitudeNums + PA.f:years + PA.f:LongitudeNums, random = ~1 |TriangleID.f.
